# Antibiotic resistance, serogroups, virulence genes, and phylogenetic groups of *Escherichia coli* isolated from yaks with diarrhea in Qinghai Plateau, China

**DOI:** 10.1186/s13099-017-0174-0

**Published:** 2017-04-28

**Authors:** Mujeeb Ur Rehman, Hui Zhang, Muhammad Kashif Iqbal, Khalid Mehmood, Shucheng Huang, Fazul Nabi, Houqiang Luo, Yanfang Lan, Jiakui Li

**Affiliations:** 1 College of Veterinary Medicine, Huazhong Agricultural University, Wuhan, 430070 People’s Republic of China; 2 Laboratory of Detection and Monitoring of Highland Animal Diseases, Tibet Agricultural and Animal Husbandry College, Linzhi, 860000 Tibet People’s Republic of China; 3 University College of Veterinary and Animal Sciences, Islamia University of Bahawalpur, Bahawalpur, 63100 Punjab Pakistan

**Keywords:** Antimicrobial sensitivity, *Escherichia coli*, Phylogeny, Serogroup, Virulence

## Abstract

**Background:**

Ruminants serve as one of the most important reservoirs for pathogenic *Escherichia coli.* Infection with *E. coli*, a foodborne enteropathogen, can lead to asymptomatic infections that can cause life-threatening complications in humans. Therefore, from a clinical and human health perspective, it is important to know which virulence genes, phylogenetic groups, serogroups, and antibiotic resistance patterns are present in *E. coli* strains in yaks with diarrheic infections.

**Methods:**

Two-hundred and ninety-two rectal swabs were collected from diarrheic yaks in Qinghai Plateau, China. The antimicrobial sensitivity of each resulting isolate was evaluated according to the disk diffusion method, and different PCR assays were performed for the detection of virulence genes and different phylogroups. Additionally, strains were allocated to different serogroups based on the presence of O antigen via the slide agglutination method.

**Results:**

Among the *E. coli* isolates tested, most of the isolates were multidrug resistant (97%) and harbored at least one virulence gene (100%). We observed ten virulence genes (*sfa, eaeA, cnf1, etrA, papC, hlyA, aer, faeG, rfc*, and *sepA*), of which *sfa* was the most commonly found (96.9%). Significant positive associations between some resistance phenotypes and virulence genes were observed (*P* < 0.05, OR > 1). The majority of the *E. coli* isolates belonged to phylogroup A (79.5%), and the others belonged to phylogroups B1 (7.5%), D (4.1%), B2 (5.8%), and F (0.7%). Among all the *E. coli* strains tested, serogroups O_91_ and O_145_ were the most prevalent, accounting for 15.4 and 14.4%, respectively.

**Conclusions:**

Our results suggest that yaks with diarrhea serve as a reservoir of pathogenic *E. coli* carrying various virulence genes and resistance phenotypes. Therefore, clinicians and relevant authorities must ensure the regulatory use of antimicrobial agents and prevent the spread of these organisms through manure to farm workers and food-processing plants.

## Background

While *Escherichia coli* is an important part of the microbiota of the intestinal tract of animals and humans, certain *E. coli* pathotypes are implicated in different animal and human infections [1, 2]. The pathogenicity of *E. coli* is determined by particular virulence traits such as capsules, toxins, invasions, adhesions, haemolysins, cytotoxic necrotic factors, and effacement factors [3]. These pathogenic *E*. *coli* are classified into intestinal (InPEC) and extraintestinal pathogenic *E. coli* (ExPEC) based on the clinical signs and virulence factors [3]. The InPEC are mainly responsible for diarrheic infections and are the leading cause of mortality especially in children. On the other hand, ExPEC are responsible for infections outside the digestive tract such as urinary tract infections, meningitis, and septicemia [1, 3].

Domestic yaks are food animals, physiologically adapted to high altitude regions of southern central Asia, Mongolia, and Russia. However, frequent outbreaks of fatal hemorrhagic diarrhea in yaks are a serious concern from both a veterinary and a human health perspective [2, 4]. As such, it is important to know, which virulence genes, phylogroups, serogroups, and antibiotic resistance patterns are present in commensal *E. coli* strains in yaks with diarrheic infections. Ruminants act as one of the most important reservoirs for pathogenic *E. coli* and lead to asymptomatic infections that can cause life-threatening complications in humans [5, 6]. Therefore, this bacterium in yaks with diarrhea may be a potential health risk if it is transmitted to humans via cross-contamination of water, food, carcasses, or feces [7].

Bacterial infections are widely treated with a variety of antibiotics in both animals and humans [8]. However, misuse of antibiotics in clinical and veterinary settings has resulted in the emergence of multidrug-resistant microbes [9, 10]. Researchers have characterized that antibiotic resistance is more common in pathogens compared to commensal organisms, and is linked to the association between resistance and virulence factors or due to frequent exposure of pathogenic strains to antibiotics [11]. However, reduced frequency of virulence determinants with high associations among resistance to certain antimicrobial agents is also reported in humans [12]. Therefore, these linkages are still not clear despite several studies. To the best of our knowledge, this is the first report examining the virulence genes, serogroups, phylogroups, and phenotypic resistance characteristics in *E. coli* strains isolated from yaks with diarrhea in China.

Currently, it is important to assess the risk of food animal-related (especially yaks) antibiotic resistance (AMR) and virulence factors on public health. Distribution of antibiotic resistance, serogroups, phylogroups, and associated virulence traits has not previously been observed in diseased or diarrheic yaks in China. This necessitates additional studies in such neglected food animals. Therefore, this study aims to characterize the possible association and distribution of phenotypes, virulence factors, phylogenetic groups, and serogroups of commensal *E. coli* strain isolated from yaks with diarrhea.

## Methods

### Sample collection, isolation, and identification

In this study, 292 rectal swabs were collected from adult yaks with diarrhea, in the Qinghai Plateau, China, between June 2015 and September 2016. Samples were collected from 37 different farms [Yushu, 20 farms (*n* = 173) and Haibei, 17 farms (*n* = 119)] from yaks raised for milk and meat purpose. Only one *E. coli* isolate was examined per rectal swab. Samples were transported to Huazhong Agricultural University (HZAU Wuhan, China) in ice-cooled containers for further experiments. All samples were enriched in nutrient broth and streaked on MacConkey agar (agar and broth media were purchased from GE Hangwei Medical Systems Co., Ltd., Beijing, China). Pink-colored colonies on MacConkey agar were subsequently used to inoculate eosin methylene blue agar (EMB), and greenish metallic-colored colonies on EMB were considered *E. coli*. Strains were then confirmed as *E. coli* via biochemical analysis, using the API 20E system (BioMerieux, Marcy-l Etoile, France; IMViC). Confirmed *E. coli* strains were suspended in Tryptic Soya Broth (TSB) and stored at −80 °C in 20% glycerol.

### Antibiotic sensitivity test

The antibiotics used in this study were based on the information provided by farm veterinarians. The antimicrobial sensitivity profile of all *E. coli* isolates was determined using the disk diffusion method, according to the criteria described by the Clinical and Laboratory Standards Institute (CLSI) [13]. Mueller–Hinton agar was used as the test medium for each of the following antimicrobials (all purchased from GE Hangwei Medical Systems Co., Ltd., Beijing, China): ampicillin (AMP, 10 µg), ceftriaxone, (CEF, 30 µg), chloramphenicol (CHP, 30 µg), ciprofloxacin (CIP, 5 µg), gentamicin (GEN, 10 µg), streptomycin (STR, 10 µg), tetracycline (TET, 30 µg), and trimethoprim/sulfamethoxazole (SXT, 1.25/23.75 µg). The test was done in triplicate for each strain and *E. coli* ATCC 25922 and *Klebsiella pneumoniae* ATCC 700603 was used as the positive and negative control strains, respectively.

### DNA extraction and screening for virulence-associated genes

Total bacterial DNA was extracted from each strain via boiling, as previously described [14], and used as the DNA template in all PCRs. All samples were then subjected to uniplex or multiplex PCR assays with specific primers for the detection of virulence genes (all the virulence genes investigated in this study were chosen based on their functional characteristics (adhesions, toxins, and capsule synthesis) and association with *E. coli* InPEC or ExPEC pathotypes, as shown in Table 1) encoding *estA, fasA, sepA* [11], *aer, cnf1* [15], *eltA, exhA, faeG, hlyA, papC, rfc, sfa* [16], *eaeA, stx1, stx2* [17], and *etrA* [18]. All testing was done with appropriate virulence genes as the positive control and sterile water as the negative control. Amplification reactions were carried out in 25 µL volumes comprising 5 µL genomic DNA, 12.5 µL 2× reaction buffer, 1 µL of each primer, 0.2 µL gold DNA polymerase, and 5.3 µL ddH_2_O using a Veriti thermal cycler (Applied Biosystems, Waltham, MA, USA). The thermocycler conditions were as follows: denaturation at 95 °C for 4 min followed by 25 cycles of denaturation at 94 °C for 30 s, variable annealing for 30 s, and extension at 72 °C for 30 s.

**Table 1:** List of 16 virulence factors screened in present study, categorized based on their association with *Escherichia coli* pathotypes

Pathotype	Virulence factor categories			Function
	Adhesins	Capsule synthesis	Toxins	
ExPEC	sfa			S fimbriae (sialic acid-specific)
			cnf1	Cytotoxic necrotizing factor 1
	papC			Pilus associated with pyelonephritis (P fimbriae)
			hlyA	α-Hemolysin
		rfc		Lipopolysaccharide synthesis
	sepA			Secreted serine protease of the auto-transporter family
EAEC	etrA			Component of ETT2 type III secretion system
EIEC	aer			Aerobatin
ETEC	faeG			F4 fimbrial adhesion
	fasA^b^			Fimbrial adhesion
			eltA^b^	Heat-labile enterotoxin
			estA^b^	Heat-stable enterotoxin
EPEC	eaeA^a^			Intimin/attaching and effacing
			exhA^a, b^	Enterohemolysin
EHEC	eaeA^a^			Intimin/attaching and effacing
			stx1^b^ stx2^b^	Shiga-toxin-I Shiga-toxin-II
			exhA^a,b^	Enterohemolysin

^a^Indicates genes shared by more than one *E. coli* pathotype

^b^Indicates none of the isolates were positive for these genes

### Phylogenetic analysis of *E. coli* strains

*Escherichia coli* strains were assigned to phylogenetic groups A, B1, B2, C, D, E, or F by quadruplex PCR analysis using a previously described protocol by targeting *chuA*, *yjaA*, *arpA,* and DNA fragment TspE4.C2 [19]. All testing was done with appropriate positive and negative controls. The results were interpreted as previously described [19].

### Serogrouping

O sero-typing was carried out using all commercially available antisera based on the slide agglutination test as per the manufacturer’s instructions (Tianjin Biochip co., LTD, Tianjin, China). For each test, polyvalent antisera and 0.5% phenol saline were also mixed together as a quality control.

### Statistical analysis

Variables were expressed as percentages (%). The significant association between antibiotic-resistant phenotypes, virulence genes, and phylogenetic groups were determined using the Pearson’s Chi-squared test. *P* values <0.05 were considered significant. Odds ratios (OR) and 95% confidence intervals were determined, and OR < 1 and >1 represent negative and positive associations, respectively. All analyses were conducted using the Stata 11 software (StataCorp Lp, College Station, TX, USA).

## Results

### Phenotypic antibiotic resistance

The highest rates of resistance were to ampicillin (95.5%), tetracycline (90.1%), and gentamicin (79.4%), with moderate rates of resistance to chloramphenicol (75.7%) and ceftriaxone (72.6%). However, 61.6, 56.8, and 43.8% of strains were resistant to trimethoprim/sulfamethoxazole, streptomycin, and ciprofloxacin, respectively (Table 2). Ninety-seven percent were MDR (resistant to at least three different classes of antibiotics), whereas 18% were resistant to all antimicrobials tested. Thirty-eight resistance patterns were observed, of which AMP-TET-GEN-CHP-CEF-SXT-STR-CIP (17.8%) was the most common. For statistical analysis, isolates of intermediate susceptibility were considered to be sensitive. Table 2 summarizes the distribution of resistance phenotypes in different phylogroups detected in *E. coli* strains isolated from yaks with diarrhea.

**Table 2:** Distribution of resistant phenotypes in different phylogroups detected in *Escherichia coli* strains isolated from yaks with diarrhea (*n* = 292)

Phylogroups (no.)	Resistant phenotypes, no.							
	AMP (*n* = 279)	TET (*n* = 263)	GEN (*n* = 232)	CHP (*n* = 221)	CEF (*n* = 212)	SXT (*n* = 180)	STR (*n* = 166)	CIP (*n* = 128)
A (*n* = 232)	223	204	195	189	166	138	132	99
B1 (*n* = 22)	22	22	17	6	17	18	14	11
B2 (*n* = 12)	12	12	3	11	10	9	7	0
D (*n* = 17)	17	17	14	14	15	13	13	9
F (*n* = 2)	2	2	1	0	2	2	0	2
ND (*n* = 7)	3	6	2	1	2	0	0	7

AMP, ampicillin; TET, tetracycline; GEN, gentamicin; CHP, chloramphenicol; CEF, ceftriaxone; SXT, trimethoprim/sulfamethoxazole; STR, streptomycin; CIP, ciprofloxacin; ND, strains that were not assigned to any phylogroup

### Prevalence of virulence genes

Ten out of 16 virulence genes were detected, namely, *sfa* (96.9%), *eaeA* (68.2%), *cnf1* (46.2%), *etrA* (24.3%), *papC* (23.3%), *hlyA* (17.5%), *aer* (12.7%), *faeG* (4.8%), *rfc* (1.7%), and *sepA* (0.7%). However, none of the isolates were positive to *stx1, stx2, exhA, eltA, estA, and fasA*. Furthermore, the *sfa* gene was most prevalent (26.7%) among the 22 virulence gene profile observed. Table 3 summarizes the distribution of virulence genes in different phylogroups and serogroups detected in *E. coli* strains isolated from yaks with diarrhea.

**Table 3:** Distribution of virulence genes in different phylogroups and serogroups detected in *Escherichia coli* strains isolated from yaks with diarrhea (*n* = 292)

Virulence gene (s)^a^	No. (%) of isolates	Phylogenetic group, no.						Serogroup, no.															
		A	B1	B2	D	F	ß	O2	O8	O52	O60	O61	O66	O91	O97	O117	O139	O145	O158	O159	O165	O172	ND^b^
	Total no.	232	22	12	17	2	7	25	13	9	5	10	11	42	18	24	5	45	10	20	8	21	26
sfa	78 (26.7)	46	12	5	10	1	4	8	6	2	1	1	3	–	11	12	3	–	6	8	–	5	12
eaeA	5 (1.7)	5	–	–	–	–	–	1	–	–	–	–	–	2	–	–	–	2	–	–	–	–	–
aer, cnf1	4 (1.4)	4	–	–	–	–	–	–	–	–	1	–	–	–	1	–	–	–	–	–	1	1	–
eaeA, sfa	63 (21.6)	51	4	3	3	1	1	3	3	2	–	–	2	22	–	3	–	6	–	–	2	11	9
aer, hlyA, sfa	3 (1.0)	3	–	–	–	–	–	–	–	–	–	–	–	1	–	–	–	2	–	–	–	–	–
cnf1, eaeA, sfa	16 (5.5)	15	1	–	–	–	–	2	–	–	1	3	–	–	–	–	–	–	–	9	1	–	–
aer, etrA, hlyA, sfa	2 (0.7)	2	–	–	–	–	–	–	–	1	–	–	–	–	–	–	–	–	1	–	–	–	–
cnf1, eaeA, etrA, sfa	45 (15.4)	45	–	–	–	–	–	2	4	2	1	4	4	6	3	5	–	5	1	–	3	1	4
cnf1, eaeA, hlyA, sfa	7 (2.4)	–	3	2	2	–	–	–	–	–	–	–	–	2	–	–	–	2	–	1	1	–	1
cnf1, eaeA, papC, sfa	9 (3.1)	8	1	–	–	–	–	2	–	–	1	–	–	–	1	3	–	–	–	1	–	1	–
faeG, hlyA, papC, sfa	2 (0.7)	1	–	–	–	–	1	–	–	–	–	–	–	–	–	–	–	2	–	–	–	–	–
faeG, hlyA, rfc, sfa	1 (0.3)	–	–	1	–	–	–	–	–	1	–	–	–	–	–	–	–	–	–	–	–	–	–
faeG, papC, sfa, sepA	1 (0.3)	1	–	–	–	–	–	–	–	–	–	–	–	–	–	–	–	–	–	–	–	1	–
papC, rfc, sfa, sepA	1 (0.3)	1	–	–	–	–	–	–	–	–	–	–	–	–	–	–	–	–	–	–	–	1	–
aer, cnf1, eaeA, papC, sfa	10 (3.4)	10	–	–	–	–	–	2	–	–	–	–	–	1	–	–	–	7	–	–	–	–	–
cnf1, eaeA, hlyA, papC, sfa	10 (3.4)	8	–	–	1	–	1	2	–	–	–	–	–	5	–	–	–	3	–	–	–	–	–
aer, cnf1, eaeA, hlyA, papC, sfa	9 (3.1)	9	–	–	–	–	–	1	–	1	–	2	2	–	–	–	–	–	2	1	–	–	–
cnf1, eaeA, etrA, hlyA, papC, sfa	15 (5.1)	15	–	–	–	–	–	–	–	–	–	–	–	1	–	–	2	12	–	–	–	–	–
cnf1, etrA, faeG, papC, rfc, sfa	1 (0.3)	–	–	–	1	–	–	–	–	–	–	–	–	–	1	–	–	–	–	–	–	–	–
eaeA, faeG, hlyA, papC, rfc, sfa	1 (0.3)	–	1	–	–	–	–	–	–	–	–	–	–	–	–	1	–	–	–	–	–	–	–
aer, cnf1, eaeA, hlyA, papC, rfc, sfa	1 (0.3)	1	–	–	–	–	–	–	–	–	–	–	–	–	–	–	–	1	–	–	–	–	–
aer, cnf1, eaeA, etrA, faeG, papC, sfa	8 (2.7)	7	–	1	–	–	–	2	–	–	–	–	–	2	1	–	–	3	–	–	–	–	–

aer, Aerobatin; cnf1, cytotoxic necrotizing factor 1; eaeA, Intimin; etrA, component of ETT2 type III secretion system; faeG, F4 fimbrial adhesion; hlyA, α-hemolysin; papC, Pilus associated with pyelonephritis (P fimbriae); rfc, lipopolysaccharide synthesis; sepA, secreted serine protease of the auto-transporter family; sfa, S fimbriae (sialic acid-specific); –, indicates none of the isolates were positive; ß, strains that were not assigned to any phylogroup; ND, strains that were not assigned to any serogroup

### Occurrence of *E. coli* pathotypes

To identify the occurrence of InPEC and ExPEC pathotypes of *E. coli*, virulence genes were grouped according to their association with different pathotypes. Overall, majority of the *E. coli* isolates carried the combinations of virulence genes, associated with both intestinal and extraintestinal pathotypes (71.2%). In addition, 27.1 and 1.7% of the isolates were positive for virulence genes associated with ExPEC and InPEC pathotypes, respectively. In the present study, all the tested virulence genes associated with ExPEC pathotype (100%) were positive for at least two isolates. Conversely, 60% of the virulence genes associated with InPEC pathotypes were not positive for a single isolate (indicated in the Table 1).

### Occurrence of phylogroups

The majority of the *E. coli* isolates belonged to phylogroup A (79.5%), with the other isolates belonging to phylogroup B1 (7.5%), B2 (4.1%), D (5.8%), and F (0.7%). However, 7 isolates (2.4%) were not assigned to any phylogenetic group. Tables 2 and 3 summarize the distributions of antibiotic resistance phenotypes, serogroups, and virulence genes among the different phylogenetic groups examined. Due to the small number of strains identified in the other phylogroups, associations between antibiotic resistance phenotypes and virulence genes were analyzed only among strains of phylogroup A (Tables 4 and 5). There was a varied statistically significant association (*P* < 0.05) between the presence of the different antimicrobial resistance phenotypes and virulence genes. Overall, a negative association was more prevalent than a positive one but the strongest positive association was detected between *cnf1/*ampicillin and *sepA/rfc* gene pairs.

**Table 4:** The associations between virulence genes and resistance phenotypes among *Escherichia coli* in phylogenetic group A (*n* = 232)

Virulence gene (*n*)	Associations of gene (OR, 95% confidence interval)^*,a^															
	No.	AMP	No.	TET	No.	GEN	No.	CHP	No.	CEF	No.	SXT	No.	STR	No.	CIP
sfa (223)	214	–	195	–	186	–	180	–	157	–	129	0.07 (0.0–1.2)	123	0.06 (0.0–0.1)	90	0.03 (0.0–0.6)
eaeA (174)	165	–	161	4.3 (1.9–9.8)	144	–	137	–	135	3.01 (1.6–5.6)	120	4.9 (2.6–9.4)	101	–	86	3.4 (1.7–6.7)
cnf1 (122)	122	22.9 (1.3–398.8)	113	2.6 (1.1–6.1)	110	2.7 (1.3–5.7)	79	0.008 (0.0–0.1)	91	–	76	–	84	2.8 (1.7–4.9)	53	–
etrA (69)	60	0.02 (0.0–0.3)	65	–	55	–	43	0.2 (0.1–0.4)	56	2.1 (1.04–4.1)	31	0.4 (0.2–0.7)	39	–	39	2.2(1.2–3.9)
papC (61)	52	0.02 (0.0–0.3)	52	–	42	0.3 (0.1–0.5)	44	0.3 (0.1-0.6)	40	–	37	–	42	2.0 (1.1–3.7)	16	0.4 (0.2–0.7)
hlyA (39)	30	0.008 (0.0–0.1)	29	0.3 (0.1–0.7)	27	0.3 (0.1–0.7)	21	0.2 (0.08–0.4)	27	–	12	0.2 (0.1-0.5)	22	–	18	–
aer (36)	36	–	26	0.3 (0.1–0.6)	24	0.3 (0.1–0.6)	14	0.08 (0.03–0.2)	22	–	33	9.5 (2.8-32.1)	26	2.2 (1.01–4.8)	11	–
faeG (9)	09	–	07	–	02	0.04 (0.0–0.2)	06	–	06	–	08	–	07	–	02	–
rfc/sepA (2)	02	–	02	–	02	–	02	–	02	–	02	–	02	–	02	–

–, indicates no significant associations (*P* ≥ 0.05); AMP, ampicillin; TET, tetracycline; GEN, gentamicin; CHP, chloramphenicol; CEF, ceftriaxone; SXT, trimethoprim/sulfamethoxazole; STR, streptomycin; CIP, ciprofloxacin

* Only antibiotic-resistant phenotypes with a significant association (*P* < 0.05) with the virulence genes in phylogenetic group A are shown

^a^Odds ratio (OR) for significant associations between genes (95% confidence interval in parenthesis)

**Table 5:** The associations between virulence genes among *Escherichia coli* in phylogenetic group A (*n* = 232)

Gene	No. (%) of isolates in phylogroup A (*n* = 232)	Associations of gene (OR, 95% confidence interval)^*,a^									
		sfa	eaeA	cnf1	etrA	papC	hlyA	aer	faeG	rfc	sepA
sfa	223	ns	**–**	**–**	8.5 (0.5–148.9)	**–**	**–**	0.2 (0.1–0.8)	**–**	**–**	**–**
eaeA	174	**–**	ns	28.4 (9.8–82.4)	17.5 (4.1–74.2)	9.2 (2.7–30.5)	**–**	**–**	**–**	**–**	na
cnf1	122	**–**	28.4 (9.8–82.4)	ns	65.8 (15.5–78.6)	32.3 (9.7–107.4)	6.4 (2.6–16.0)	7.1 (2.71–9.2)	**–**	**–**	na
etrA	69	8.5 (0.5–148.9)	17.5 (4.1–74.2)	65.8 (15.5–278.6)	ns	**–**	2.1 (1.0–4.2)	**–**	9.1 (1.8–44.9)	na	na
papC	61	**–**	9.2 (2.7–30.5)	32.3 (9.7–107.4)	**–**	ns	41.8 (15.0–116.3)	14.3 (6.2–33.1)	62.1 (3.5–1084.4)	14.4 (0.7–304.5)	14.4 (0.7–304.5)
hlyA	39	**–**	**–**	6.4 (2.6–16.0)	2.1 (1.0–4.2)	41.8 (15.0–116.3)	ns	5.1 (2.3–11.3)	**–**	**–**	na
aer	36	0.2 (0.1–0.8)	**–**	7.1 (2.7–19.2)	**–**	14.3 (6.2–33.1)	5.1 (2.3–11.3)	ns	23.4 (4.6–118.2)	**–**	na
faeG	9	**–**	**–**	**–**	9.1 (1.8–44.9)	62.1 (3.5–1084.4)	**–**	23.4 (4.6–118.2)	ns	na	27.7 (1.6–484.6)
rfc	2	**–**	**–**	**–**	**–**	14.4 (0.7–304.5)	**–**	**–**	**–**	ns	229 (7.7–684.3)
sepA	2	**–**	**–**	–	**–**	14.4 (0.7–304.5)	**–**	**–**	27.7 (1.6–484.6)	229 (7.7–684.3)	ns

–, indicates no significant associations (*P* ≥ 0.05); na, no results available (OR could not be calculated because none of the isolates carried one of the combinations of virulence genes or the value of one of the genes was a constant or zero); ns, no statistics were determined

* Only virulence genes with a significant association (*P* < 0.05) are shown

^a^Odds ratio (OR) for significant associations between virulence genes (95% confidence interval in parenthesis)

### Occurrence of serogroups

The distribution of serotypes varied in *E. coli* strains isolated from yaks with diarrhea. Of the *E. coli* strains tested, serogroups O_91_ and O_145_ were the most prevalent, accounting for 15.4 and 14.4%, respectively. Lastly, 8.9% of the isolates were untyped with the available antisera as shown in Table 2.

## Discussion

In present study, we observed a higher proportion of multidrug-resistant *E. coli* with virulence factors in yaks suffering from diarrheic infections, and determined the correlations among virulence genes and resistance phenotypes. These data can be compared to the reports of other regions and in other animals since the study of antimicrobial resistance in important food animals such as yak is still inadequate.

In this study, ninety-seven percent of the *E. coli* isolates were resistant to at least three different classes of antibiotics (MDR), whereas 18% were resistant to all antimicrobials tested. The phenotypic resistance to ampicillin and tetracycline was identified at a high rate, similar to the previous findings in the isolates from diarrheic or diseased animals in China [17, 20–23]. The predominance of tetracycline resistance among the *E. coli* strains from diarrheic yaks was similar to the findings of Boerlin et al. [11], who detected tetracycline resistance in 96 out of 100 *E. coli* strains isolated from diarrheic pigs. However, similar levels of resistance were observed in *E. coli* strains isolated from healthy pigs and chickens [24]. Altogether, these findings reflect the widespread and heavy use of such antibiotics in animals in China. Approximately 30% of drugs sold in China are antibiotics, which is 20% higher than the proportion in the developed world [25]. Furthermore, China has the highest rate of antimicrobial resistance (enteric gram-negative bacilli) in both community and hospital-acquired infections among Asian courtiers, along with Singapore and Philippines [26]. As such, antimicrobial resistance is a major public health concern in China [27]. The use of chloramphenicol in food animals is banned, as there is a high frequency of chloramphenicol-resistant phenotypes. The high levels of chloramphenicol resistance have been formerly reported in other bacteria of animal origin and are probably linked to the proficient horizontal dissemination of resistance determinants or co-selection of resistant genes [28]. Furthermore, the high resistance to antibiotics in the study area may be a sign of difference in disease control practices, antimicrobial usage, or various unknown factors such as genetic mutations contributing towards multidrug-resistant phenotypes [29]. Therefore, strong surveillance programs are needed to control the dissemination of antibiotic resistance in nomadic pastorals of China like Qinghai–Tibetan Plateau.

In present study, various virulence genes were identified in the *E. coli* isolates, suggesting the existence of pathogenic *E. coli* in yaks with diarrhea. Overall, our results showed that a significant fraction of *E. coli* isolates from diarrheic yaks are possible diarrheagenic and extraintestinal pathotypes. It is particularly worrisome that all (100%) the tested virulence genes, associated with ExPEC pathotype (100%) were positive for at least two isolates. Conversely, 60% of the InPEC pathotype did not reveal any associated virulence gene under investigation. This observation indicates a high potential health concern as virulence genes associated with ExPEC pathotype were more common in diarrheic yaks, which is considered to be a possible health risk due to their pathogenic potential [3]. Moreover, our results present a possibility that the observed combinations of virulence genes are involved in a distinct category of diarrheagenic *E. coli.* Nevertheless, the occurrence of single or multiple virulence factors in an *E. coli* strain does not essentially signify that a strain is pathogenic because *E. coli* uses a multifaceted mechanism of pathogenesis [30, 31]. Therefore, further studies in animal model or tissue culture are required to demonstrate the pathogenicity of observed virulence genes/patterns.

In addition, we observed a moderate number of virulence-associated genes of both InPEC and ExPEC categories in diarrheic yaks. This could be explained by the harsh environmental conditions at Qinghai–Tibetan Plateau (average altitude 3000 m). Yaks are physiologically adapted to high altitude environmental conditions (hypoxia, pH, and high altitude radiations) that genetically equip them with relatively stronger ability of resistance or tolerance to infections [32, 33]. In present study, *sfa* and *eaeA* were the most abundant virulence genes, which are linked with ExPEC and InPEC pathotypes, respectively. Conversely, all isolates were negative for the InPEC-associated toxin genes (*stx1, stx2, exhA, eltA, and estA),* but positive for ExPEC-associated toxin genes (*cnf1* and *hly*A). Such observation has not been commonly described in previous reports of *E. coli* isolates of animal origin [2, 7, 10, 17]. This observed variation could be attributed to the existing climatic conditions which may account for the diverse occurrence of virulence-associated genes. Furthermore, we also observed that a relatively high number of *E. coli* isolates carried a combination of *sfa and eaeA* genes. The exact significance of this combination is not clear. However, the *eaeA* gene is involved in adherence to epithelial cells [producing characteristic attaching and effacing (A/E) lesions] [34] and *sfa* may possibly assist with the fixation of *E. coli* in the gastrointestinal tract of yaks. Further studies are required to understand this phenomenon. In addition, the occurrence of unusual patterns of virulence genes observed in current study might be due to horizontal gene transfer (integrons, plasmids, transposons) between related or unrelated bacterial species [35].

The association among virulence genes and resistance phenotypes varied in this study. Overall, negative correlations were more common between virulence genes and phenotypic resistance. We observed the strongest association between ExPEC-associated toxin gene (*cnf1*) and ampicillin. In addition, EHEC-associated adhesion gene (*eaeA*) was the most prevalent associated gene with the resistance to tetracycline, ceftriaxone, ciprofloxacin, and trimethoprim/sulfamethoxazole. Aerobain (*aer*) and component of ETT2 type III secretion system (*etrA*) were also significantly associated with the resistance to ceftriaxone, ciprofloxacin, and streptomycin, trimethoprim/sulfamethoxazole, respectively. Furthermore, resistance to streptomycin was significantly associated with increased frequency of *papC,* and *cnf1*. Such associations were not observed in previous studies [10, 15, 21]. Our findings suggest that the association of virulence and resistance might be strain-specific or due to various antibiotics used in different geographical regions. Notably, negative correlations were not observed among virulence genes (except *aer* and *sfa* gene pairs), and strongest associations were observed among virulence genes, *sepA* and *rfc* gene pairs followed by *etA*/*cnf1* and *faeG/papC* gene pairs. Such associations were not observed in previous studies [10, 15, 21]. Taken together, these findings suggest that associations among resistance and virulence genes in *E. coli* isolates vary with antimicrobial use and to a certain phylogenetic background. Moreover, we examined only the phenotypic profile of our isolates (in vitro). Therefore, further studies are required to elaborate the real significance of the observed associations and its impact on different outcomes of infection.

All the *E. coli* strains were allocated to phylogenetic groups, A, B1, B2, C, D, E, and F as previously suggested [19]. Based on the phylogenetic analysis, antibiotic-resistant *E. coli* isolates of animal origin were not associated with low virulence traits [12, 36]. It has been reported that ExPEC, a prominent zoonotic infection that is responsible for urinary tract infections in humans, is mainly associated with groups B2 and D [37]. Especially, extraintestinal virulence is considered to be epidemiologically linked with the phylogroup B2 by means other than the known extraintestinal virulence factors [38]. In contrast, groups A and B1 were reported to be associated with InPEC and commensal strains [39]. In this study, the majority of the *E. coli* isolates belonged to group A (79.5%) and the remaining to phylogroups B1, B2, D, or F. Moreover, *E. coli* adhesion gene (*sfa*) was the most common gene observed in phylogroup A strains. The exact significance of this combination is not clear. However, it suggests a possible role of this combination (phylogroup A and adhesion gene, *sfa*) in the diarrheic infections, as these strains were obtained from yaks with diarrhea. Furthermore, the high prevalence of phylogroup A identified was consistent with the appurtenance of the isolates examined in this study, and our findings were in line to those noted in some previous studies of diarrheagenic and commensal strains [40, 41].

We observed 15 different O serotypes among the diarrheic yaks. Interestingly, we found that O91 and O145 had the highest frequency of virulence genes. STEC serogroup O157 and non-O157 strains such as O26, O91, O103, O111, O113, O128, O121, and O145 have been shown to cause diarrhea [42, 43]. The high prevalence of O91 and O145 identified was inconsistent with the findings of previous reports from patients with diarrhea [44, 45]. Serogroups O2, O8, O60, O61, O66, O91, O97, O117, O158, O159, O165, and O172 were earlier identified in humans and animals with diarrhea [2, 6, 46, 47]. However, the serogroups O2 and O60 were also reported in MDR uropathogenic isolates of *E. coli* from patients with prostatitis, simple UTI, pyelonephritis, and cystitis in India [29]. Lastly, the serogroups O52 and O139 detected in this study appear to be additional serotypes associated with diarrhea in yaks. If these yaks do not receive effective treatment, they are prone to secondary infections and diseases.

## Conclusion

The findings of the present study highlight the important role of yaks as a potential reservoir of drug-resistant *E. coli* with a variety of virulent determinants that may spread into the environment and to humans. The association between resistance and virulence genes sustains the concerns that virulence traits in yaks can be selected by antibiotic usage in the farms. Therefore, we recommend that strong surveillance programs be initiated to control and monitor the frequency and regulatory use of antimicrobial agents.
